# Supplementary material for: Degeneracy in epilepsy: multiple routes to hyperexcitable brain circuits and their repair
Source: Commun Biol. 2023 May 3;6:479. doi: 10.1038/s42003-023-04823-0 (PMC10156698; doi:10.1038/s42003-023-04823-0)
Supplement: Supplementary file 2 — Description of Additional Supplementary Files [file 42003_2023_4823_MOESM2_ESM.pdf]

## Description of Additional Supplementary Files

**File Name:** Supplementary Data 1
**Description:** Source data underlying Figure 2a.
