# Supplementary material for: Degeneracy in epilepsy: multiple routes to hyperexcitable brain circuits and their repair
Source: Commun Biol. 2023 May 3;6:479. doi: 10.1038/s42003-023-04823-0 (PMC10156698; doi:10.1038/s42003-023-04823-0)
Supplement: Supplementary file 3 — Supplementary Data 1 [file 42003_2023_4823_MOESM3_ESM.pdf]

Number of nsSNPs in hEP Genes	Numer of control individuals	Number of affected individuals
0	46	6
1	52	28
2	13	43
3	13	33
4	9	18
5	2	15
6	1	6
7	1	0
8	0	0
9	0	1
10	0	0
Data manually extracted from Klassen et al., 2011, Cell, Figure 3C		
